# Supplementary material for: Resistance to Triazoles in Populations of Mycosphaerella fijiensis and M. musicola from the Sigatoka Disease Complex from Commercial Banana Plantations in Minas Gerais and São Paulo, Brazil
Source: Microorganisms. 2025 Jun 20;13(7):1439. doi: 10.3390/microorganisms13071439 (PMC12300740; doi:10.3390/microorganisms13071439)
Supplement: Supplementary file 1 [file microorganisms-13-01439-s001.zip › Supplementary file S2.pdf]

Supplementary file S2. Analysis of variance of the effects of species, geographical populations, CYP51 variants, and isolates in the levels of sensitivity to DMI fungicides propiconazole and tebuconazole in *Mycosphaerella fijiensis* and *M. musicola* from banana plantations in São Paulo and Minas Gerais, Brazil *.

**Fungicide: Propiconazole**						
**Source of variation**	**Levels**	**Degrees of freedom (Df)**	**Sum of square (Sum Sq)**	**Mean Sq**	***F* value**	***p* > F**
Isolates	51	50	12.528	0.251	153,703	$\leq 2^{-16}$ ***
Residuals		357	0.001	0.000		
Protein variants	8	7	6.303	0.901	57.86	$\leq 2^{-16}$ ***
Residuals		400	6.226	0.016		
Populations	4	3	5.822	1.941	115.9	$\leq 2^{-16}$ ***
Residuals		404	6.707	0.017		
Species	2	1	5.792	5.792	349.1	$\leq 2^{-16}$ ***
Residuals		406	6.737	0.017		
Fungicide: Tebuconazole						
**Source of variation**	**Levels**	**Degrees of freedom (Df)**	**Sum of square (Sum Sq)**	**Mean Sq**	***F* value**	***p* > F**
Isolates	51	50	49.07	0,981	16,781	$\leq 2^{-16}$ ***
Residuals		357	0.02	0.0001		
Protein variants	8	7	34.540	4.935	135.7	$\leq 2^{-16}$ ***
Residuals		400	14.550	0.036		
Populations	4	3	29.910	9.969	210.0	$\leq 2^{-16}$ ***
Residuals		404	19.180	0.047		
Species	2	1	29.220	29.218	569.9	$\leq 2^{-16}$ ***
Residuals		406	19.870	0.049		

*The species effect compared *M. fijiensis* (N=9) and *M. musicola* (N=42); the population effect compared four geographical populations MGN-C (N=15), SPNW-C(N=13), SPNW-O (N=14) from *Mm*, and SPVR-CI (N=9) from *Mf*; the protein variant effect compared eight variants [A (N=31), B (N=2), C (N=3), D (N=1), E (N=5), F (N=5), G (N=3), and H (N=1)] detected in the fungal populations sampled; N = number of isolates within each factor.
